# Differences in disease severity and prognosis of exercise-induced right-to-left shunt between idiopathic pulmonary arterial hypertension and chronic thromboembolic pulmonary hypertension patients

**DOI:** 10.3389/fcvm.2022.976730

**Published:** 2022-12-12

**Authors:** Shang Wang, Zi-Yan Guo, Xing-Xing Sun, Ping Yuan, Qin-Hua Zhao, Wen-Hui Wu, Hong-Ling Qiu, Ci-Jun Luo, Su-Gang Gong, Hui-Ting Li, Rui Zhang, Jing He, Lan Wang, Jin-Ming Liu, Jian Guo, Rong Jiang

**Affiliations:** ^1^Department of Cardiopulmonary Circulation, Shanghai Pulmonary Hospital, School of Medicine, Tongji University, Shanghai, China; ^2^Department of Cardiovascular Intensive Care Unit, The First Affiliated Hospital of Henan University, Kaifeng, Henan, China; ^3^Department of Pulmonary Function Test, Shanghai Pulmonary Hospital, School of Medicine, Tongji University, Shanghai, China

**Keywords:** pulmonary hypertension, cardiopulmonary exercise test, venous-to-systemic shunt, right heart catheterization, idiopathic pulmonary arterial hypertension, chronic thromboembolic pulmonary hypertension

## Abstract

**Objective:**

Whether exercise-induced venous-to-systemic shunt (EIS) during cardiopulmonary exercise testing (CPET) has different manifestations or characteristics in idiopathic pulmonary arterial hypertension (IPAH) and chronic thromboembolic pulmonary hypertension (CTEPH) patients remains unknown. We explored the differences in hemodynamics, echocardiography, and prognosis between IPAH and CTEPH patients with and without EIS.

**Methods:**

We conducted a retrospective cross-sectional cohort study and included 161 PH patients at Shanghai Pulmonary Hospital. Demographic, echocardiography, pulmonary hemodynamic, and CPET variables were compared between patients with and without EIS stratified by IPAH and CTEPH. EIS was determined by CPET. Binary logistic regression analyses were performed to explore independent influencing factors of EIS. Cox survival analysis was used to quantify the impact of EIS on the prognosis of patients.

**Results:**

Exercise-induced venous-to-systemic shunt was found in approximately 17.4% of 86 IPAH patients and 20% of 75 CTEPH patients. All-cause mortality occurred in 43 (26.7%) patients during a median follow-up of 6.5 years. Compared with those without EIS, patients with EIS had higher peak end-tidal O_2_ and lower VO_2_/VE and tricuspid annular plane systolic excursion (TAPSE). Among the IPAH patients, EIS was associated with lower cardiac output, cardiac index, mixed venous oxygen saturation, VO_2_/VE, and TAPSE and higher VE/VCO_2_ and right ventricular end-diastolic transverse diameter. Logistic regression analysis indicated that VO_2_/VE was an independent factor influencing whether IPAH patients developed EIS during CPET. Cox logistic regression indicated that female IPAH patients or IPAH patients with higher VO_2_/VE and EIS had a better prognosis. Female IPAH patients had better 10-year survival. In IPAH patients without EIS, patients with higher VO_2_/VE had better 10-year survival. However, compared with CTEPH patients without EIS, those with EIS had similar echocardiographic, hemodynamic, CPET parameter results and 10-year survival.

**Conclusion:**

Exercise-induced venous-to-systemic shunt exhibits different profiles among IPAH and CTEPH patients. Among IPAH patients, those with EIS had worse peak end-tidal O_2_, VO_2_/VE, and TAPSE than those without EIS. VO_2_/VE was an independent factor of EIS among IPAH patients. IPAH patients with EIS, female sex or higher VO_2_/VE had better survival. However, the association between EIS and PAH severity or prognosis in CTEPH patients needs to be further explored.

## Introduction

Pulmonary hypertension (PH) is a progressive and fatal disease that is characterized by an increase in pulmonary vascular resistance (PVR) and progressive structural remodeling of pulmonary arteries, ultimately leading to right ventricular failure and death (1, 2). According to clinical classification, it is divided into GROUP 1–5 PH (3). Pulmonary arterial hypertension (PAH) and chronic thromboembolic pulmonary hypertension (CTEPH) are GROUP 1 PH and GROUP 4 PH, respectively. CTEPH is caused by pulmonary vasculature obstruction and has a high mortality (4, 5). In addition, it is well-established that PAH is associated with abnormal cardiopulmonary exercise testing (CPET), such as decreased exercise tolerance, ventilation efficiency, and cardiac function and submaximal exercise tolerance (6–8). Changes in gas exchange patterns caused by exercise-induced right-to-left shunt (EIS) through the patent foramen ovale (PFO), which occurs during CPET, are frequently observed in PAH patients (9). The incidence of EIS in PAH patients is approximately 40% (7, 9). However, the relationship between EIS and prognosis in patients with PH is currently unclear. Some researchers have suggested that chronic PH increases the potential for shunting through the foramen ovale and that such shunting may favor increased survival (9). Nevertheless, others contend that the persistence or development of EIS strongly predicts death or transplantation independent of hemodynamics and all other exercise measures (10).

In the fetal stage, the foramen ovale is a physiological and anatomical structure required to maintain the normal development of the embryo. As the pressure of the left atrium gradually exceeds that of the right atrium, the foramen ovale generally undergoes functional closure before the first year of life (11). The PFO is found in approximately 25% of the adult population and can cause a significant right-to-left shunt when performing things that raise the thoracic pressure, such as Valsalva movements and coughing (11–13). With regard to PAH patients, the increase in exercise load further leads to an increase in PVR because flow-mediated dilatation of the lung circulation cannot increase appropriately, which eventually leads the right atrial pressure (RAP) to exceed the left atrial pressure, and then EIS occurs. The presence of an EIS can cause venous blood with low PaO_2_, high PaCO_2_ and H^+^ to enter the systemic circulation directly through the PFO, which stimulates the systemic circulation arterial chemoreceptors and causes hyperventilation of the lung (14).

The current literature on EIS during CPET has focused on WHO Group 1 PH, especially in idiopathic pulmonary arterial hypertension (IPAH) patients. The presence of EIS has proven to be associated with the severity of exercise limitation and poor hemodynamics and prognosis in patients with IPAH (7, 9, 10). We have previously compared the differences in CPET between IPAH and CTEPH patients. The oxygen uptake efficiency plateau and oxygen uptake efficiency at the anaerobic threshold are higher in IPAH patients than in CTEPH patients and are not in proportion to hemodynamics, probably due to differences in pulmonary vascular occlusion (15). In addition to microarterial lesions similar to those in IPAH patients, CTEPH patients have thrombi in the main trunk, leaves, segments, subsegments and other parts. In addition, whether there are EIS differences between IPAH and CTEPH patients or whether EIS is associated with severity and prognosis in CTEPH patients remains unknown.

Therefore, our purpose was (1) to compare the different profiles of the hemodynamics, echocardiography and CPET in PH patients and subpopulations with or without EIS; (2) to explore the risk factors associated with EIS; and (3) to assess the impact of EIS on the prognosis of IPAH and CTEPH patients.

## Materials and methods

### Study participants and design

**FIGURE 1:**
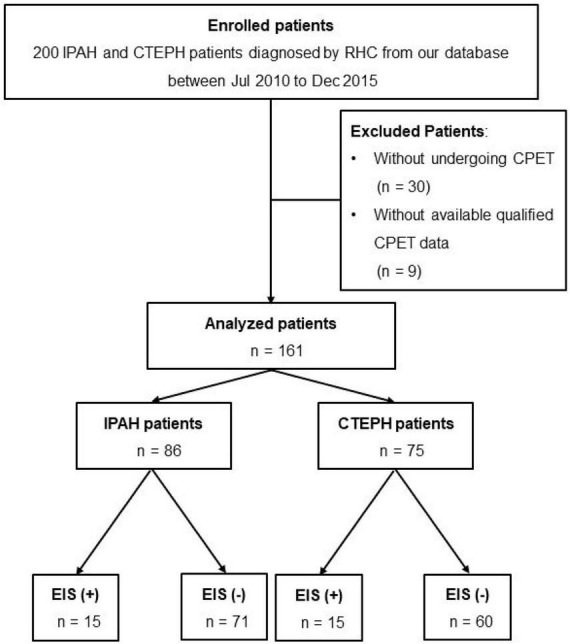
Flow diagram for the cohort. CTEPH, chronic thromboembolic pulmonary hypertension; IPAH, idiopathic pulmonary arterial hypertension; RHC, right heart catheterization; CPET, cardiopulmonary exercise testing.

A total of 200 patients diagnosed with IPAH or CTEPH from 2010 to 2015 were screened from our center database, and 161 patients with available CPET results were finally included (Figure 1). The diagnosis of IPAH and CTEPH was based on the diagnostic criteria of PH (16, 17). The exclusion criteria were (1) patients with other causes of PH, such as chronic lung disease or left heart disease-associated PH (16); (2) patients who received pulmonary endarterectomy (PEA) or balloon pulmonary angioplasty (BPA) treatment; and (3) patients with no right heart catheterization (RHC) or CPET records. RHC was performed within 1 week of each patient’s CPET examination. Demographic information, 6-min walk distance (6MWD), WHO functional class, echocardiographic parameters, PAH risk score (low, intermediate, or high), and hemodynamic features were determined at baseline during hospitalization. This study was conducted in accordance with the amended Declaration of Helsinki. The Institutional Ethics Committee of Shanghai Pulmonary Hospital approved the protocol.

### Hemodynamic measurements

Right heart catheterization was performed in all patients during hospitalization. RHC was performed as described previously (18, 19). Mean pulmonary arterial pressure (mPAP), right atrial pressure (RAP), pulmonary arterial wedge pressure (PAWP), and cardiac output (CO) were measured. CO was measured in triplicate by the thermodilution technique with iced normal saline. The cardiac index (CI) was calculated by dividing CO by body surface area. Pulmonary vascular resistance (PVR) was calculated by dividing the mPAP minus the PAWP by CO.

### Cardiopulmonary exercise test measurements

All the patients and controls exercised on a cycle ergometer using a breath-by-breath system according to the American Thoracic Society/American College of Chest Physicians statement on CPET (20). Before each test, the equipment was calibrated in accordance with the manufacturer’s specifications using reference and calibration gases. The protocol comprised 3 min of rest, 3 min of unloaded cycling at 55–65 revolutions per minute (rpm), followed by a progressively increasing work rate of 5–15 watts (W)/min for PH patients to the maximum tolerance, and 4 min of recovery (8, 21). Direct measurements of oxygen uptake ($\dot{\text{V}}\,$O_2_), carbon dioxide output ($\dot{\text{V}}\,$CO_2_), minute ventilation ($\dot{\text{V}}\,$E), end-tidal CO_2_ (P_ET_CO_2_), end-tidal O_2_ (P_ET_O_2_) and several derived parameters, such as the heart rate (HR), oxygen pulse ($\dot{\text{V}}\,$O_2_/HR), and respiratory exchange ratio (RER), were obtained. Peak $\dot{\text{V}}\,$O_2_ was defined as the highest 30-s average of oxygen uptake in the last minute of exercise, and other peak parameters were calculated at the same time. The $\dot{\text{V}}\,$E-$\dot{\text{V}}\,$CO_2_ slope was determined by linear regression analysis of the relationship between $\dot{\text{V}}\,$E and $\dot{\text{V}}\,$CO_2_ during exercise, excluding data above the ventilatory compensation point (22). The lowest $\dot{\text{V}}\,$E/$\dot{\text{V}}\,$CO_2_ was determined by averaging the lowest consecutive 90-s data points (22). $\dot{\text{V}}\,$O_2_/$\dot{\text{V}}\,$E, known as oxygen uptake efficiency (OUE), was computed by linear least squares regression of the oxygen uptake on the logarithm of the minute ventilation according to the following equation: $\dot{\text{V}}\,$O_2_ = a × log $\dot{\text{V}}\,$E + b, where the constant *a* is the OUE (7, 8, 23). The oxygen uptake efficiency plateau (OUEP) was the highest 90-s stretch of $\dot{\text{V}}\,$O_2_ (mL/min)/$\dot{\text{V}}\,$E (L/min) (7, 8, 23).

### Detection of exercise-induced venous-to-systemic shunt by gas exchange criteria

EIS was determined by two investigators who were blinded to all the patients’ clinical courses. Nine-panel CPET plots were independently reviewed to identify EIS during exercise using the following criteria as described in detail previously: an abrupt and sustained increase in P_ET_O_2_, RER, $\dot{\text{V}}\,$E/$\dot{\text{V}}\,$O_2_, and $\dot{\text{V}}\,$E/$\dot{\text{V}}\,$CO_2_ with a simultaneous, sustained decrease in P_ET_CO_2_ and pulse oximetry (SpO_2_) (9).

### Separation of PH patients into groups

Using the above criteria, two investigators independently graded the 161 PH patients as either EIS positive (+) or EIS negative (−). Patients who were graded consistently as EIS (+) by all three graders were placed in the shunt group, and those who were graded consistently as EIS (−) by all three graders were placed in the no-shunt group.

### Echocardiography

The process of transthoracic echocardiography was as previously described (24, 25). Right ventricular end-diastolic transverse dimension (RVEDTD), right ventricular end-diastolic longitudinal dimension (RVEDLD), right atrial transverse dimension (RATD), right atrial longitudinal dimension (RALD), end-systolic stage left ventricular eccentricity index (ENDSEI), pulmonary arterial systolic pressure (PASP), and tricuspid annular plane systolic excursion (TAPSE) were measured.

### Follow-up

Clinical, demographic, echocardiographic, CPET, and RHC parameters were collected from hospital records. The primary endpoint was all-cause mortality. Mortality information during follow-up was obtained from chart review, outpatient clinic visits or telephone interviews. The follow-up lasted from the date of CPET until the patient died or was censored at the end of the study (31 January 2021).

### Statistical analysis

Data are described as the mean ± standard deviation or median and interquartile and counts (proportions), as appropriate. Spearman’s or Pearson’s correlation coefficient was calculated, as appropriate. Binary logistic regression was applied to find the variables associated with EIS (+) among the IPAH or CTEPH patients separately. Univariate and multivariate Cox proportional hazards regression analyses were applied to quantify the relationships between all-cause mortality and variables of interest. After correction by collinearity analysis, the variables significant in univariate analysis were included in the multivariate Cox regression model. Wald chi-square statistics and 95% confidence intervals (CIs) were used to calculate the significance of the estimates at the level of 0.05. Event-free survival was estimated from the time of CPET, with all-cause mortality as the endpoint. The best separation cutoff values of the parameters, as judged from the log-rank χ^2^ statistics, were identified by the *survminer* package of R software. Kaplan–Meier survival curves and log-rank tests were used to assess EIS and other variables for survival outcomes. Statistical analysis was performed using R software (version 4.1.3).^1^ In all univariate analyses, *P* < 0.05 was considered statistically significant.

## Results

### Study population

None of the 200 patients ultimately enrolled in this study had discordant evaluations. The medical records of 161 patients with IPAH (*n* = 86) or CTEPH (*n* = 75) who systematically underwent RHC and CPET for clinical evaluation at Shanghai Pulmonary Hospital between January 2010 and December 2015 were retrospectively studied (Figure 1). Among them, 15 IPAH and 15 CTEPH patients had EIS. The PFO was found in approximately 14.7 and 20% of IPAH and CTEPH patients, respectively. A comparison between demographic, hemodynamic, CPET, and echocardiography characteristics was made between EIS (+) and EIS (−) patients. Compared with EIS (−) PH patients, EIS (+) PH patients had worse peak P_ET_O_2_, VO_2_/VE, and TAPSE despite having comparable pulmonary hemodynamics (Table 1). These results indicate that EIS (+) PH patients had impaired ventilation efficiency and submaximal exercise tolerance and right ventricular function (Table 1). All echocardiographs were performed at rest, and the PFO was found by echocardiography at rest. Table 2 shows the detailed comparisons of hemodynamics, CPET and echocardiography, stratified by PH category, namely, IPAH and CTEPH.

**TABLE 1:** Demographic, hemodynamic, cardiopulmonary exercise testing, and echocardiographic data in patients with or without EIS.

Variable	EIS (+) (*n* = 30)	EIS (−) (*n* = 131)	*P*-value
Age, years	47.3 ± 15.9	48.2 ± 16.9	0.794
Female, n (%)	22 (73.3)	90 (68.7)	0.667
PH classification, n (%)			0.690
IPAH	15 (50.0)	71 (54.2)	
CTEPH	15 (50.0)	60 (45.8)	
WHO FC, n (%)			0.347
II	11 (36.7)	65 (49.6)	
III	18 60.0)	63 (48.1)	
IV	1 (3.3)	3 (2.3)	
BMI, kg/m^2^	23.7 ± 3.8	22.7 ± 2.8	0.107
Risk classification, n (%)			0.263
Low	8 (26.7)	55 (42.0)	
Moderate	19 (63.3)	63 (48.1)	
High	3 (10.0)	13 (10.0)	
Status, n (%)			0.652
Survivors	21 (70.0)	97 (74.0)	
No survivors	9 (30)	34 (26.0)	
**Right heart catheterization**			
mPAP, mmHg	47.8 ± 14.5	50.1 ± 13.7	0.421
PAWP, mmHg	7.0 (4.8, 10.0)	7.0 (5.0, 9.0)	0.884
CO, L/min	4.6 ± 1.4	4.8 ± 1.2	0.457
CI, L/min/m^2^	2.7 ± 0.7	2.9 ± 0.7	0.220
mRAP, mmHg	5.0 (5.0, 8.0)	8.0 (5.0, 8.0)	0.416
PVR, mmHg	9.7 (5.6, 13.3)	8.3 (6.2, 12.1)	0.313
SvO_2_, %	65.5 (57.7, 69.0)	66.2 (61.0, 70.7)	0.163
**Cardiopulmonary exercise testing**			
Peak VO_2_/kg, mL/min/kg	12.6 (9.8, 15.3)	13.7 (11.2, 16.1)	0.377
Peak P_ET_O_2_, mmHg	129.4 ± 6.1	126.0 ± 6.8	**0.012**
Peak P_ET_CO_2_, mmHg	22.8 ± 7.0	24.5 ± 7.8	0.272
peak VO_2_/HR, L/min	5.5 (4.1, 6.6)	5.8 (4.8, 6.9)	0.122
RER	1.1 (1.0, 1.2)	1.1 (1.0, 1.1)	0.056
VO_2_/VE ml/L	16.7 ± 4.6	19.7 ± 5.1	**0.004**
VE/VCO_2_ slope (%pred)	54.0 (42.6, 65.8)	49.2 (40.8, 59.5)	0.084
OUEP, ml/L	24.3 ± 4.5	25.6 ± 5.3	0.240
Lowest VE/VCO_2_ (%pred)	46.5 (38.6, 56.5)	44.5 (38.8, 53.4)	0.564
**Echocardiography**			
TAPSE, cm	1.6 (1.4, 1.9)	1.7 (1.6, 2.0)	**0.041**
RATD, cm	4.6 ± 1.1	4.5 ± 0.9	0.547
RALD, cm	5.1 (4.4, 5.8)	5.1 (4.6, 5.8)	0.717
RVEDTD, cm	4.3 (3.6, 4.8)	4.3 (3.5, 4.5)	0.198
RVESTD, cm	6.5 ± 0.8	6.4 ± 0.9	0.591
ENDSEI	1.3 (1.1, 1.5)	1.2 (1.0, 1.4)	0.176
PASP, mmHg	67.0 (56.8, 89.2)	70.0 (56.0, 91.0)	0.879
Targeted-PAH treatment			0.204
Ambrisentan + Sildenafil	7 (23.3)	53 (40.5)	
Ambrisentan + Bosentan + Tadalafil	13 (43.3)	47 (35.9)	
Macitentan + Riociguat + Tadalafil	10 (33.3)	31 (23.7)	

Values are expressed as the mean ± SD, n (%), or median (interquartile range) and percentage of measured to predicted values (%pred). BMI, body mass index; CI, cardiac index; CO, cardiac output; CTEPH, chronic thromboembolic pulmonary hypertension; EIS, exercise-induced venous-to-systemic shunting; ENDSEI, end-systolic stage eccentricity index; EIS, exercise-induced right-to-left shunt; IPAH, idiopathic pulmonary arterial hypertension; mPAP, mean pulmonary artery pressure; mRAP, mean right atrial pressure; OUEP, oxygen uptake efficiency plateau; PASP, pulmonary arterial systolic pressure; PAWP, pulmonary artery wedge pressure; peak P_ET_CO_2_, peak end-tidal partial pressure of CO_2_; peak P_ET_O_2_, peak end-tidal partial pressure of O_2_; peak VO_2_/HR, peak oxygen pulse; peak VO_2_/kg, peak oxygen consumption per kilogram; PVR, pulmonary vessel resistance; RALD, right atrial longitudinal dimension; RATD, right atrial transverse dimension; RER, respiratory exchange ratio; RVEDLD, right ventricular end-diastolic longitudinal dimension; RVEDTD, right ventricular end-diastolic transverse diameter; SvO_2_, mixed venous oxygen saturation; TAPSE, tricuspid annular plane systolic excursion; VCO_2_, carbon dioxide output; VE, minute ventilation; VE/VCO_2_ slope, the slope between VE and VCO_2_; VO_2_, oxygen uptake; VO_2_/VE slope, the slope between VO_2_ and VE; WHO FC, World Health Organization functional class. *P* < 0.05 is represented by bold values.

**TABLE 2:** Comparisons of demographic, hemodynamic, cardiopulmonary exercise testing, and echocardiographic variables in IPAH or CTEPH patients with vs. without EIS.

Variable	IPAH		CTEPH	
	EIS (+)	EIS (−)	EIS (+)	EIS (−)
	(*n* = 15)	(*n* = 71)	(*n* = 15)	(*n* = 60)
Age, years	36.5 ± 10.4^b^	38.8 ± 14.6^c^	58.0 ± 13.0	59.2 ± 12.0
Sex, female/male	4/11	52/19	11/4	22/38
**WHO FC, n (%)**				
II	5 (33.3)	23 (32.4)	6 (40.0)	22 (36.7)
III	10 (66.7)	27 (38.0)	8 (53.3)	36 (60)
IV	0 (0.0)	1 (0.0)	1 (0.1)	2 (0.0)
BMI, kg/m^2^	22.4 ± 3.2	22.4 ± 3.0	25.0 ± 4.0	23.0 ± 2.6
**Risk classification, n (%)**				
Low	2 (13.3)	34 (47.8)	6 (40.0)	21 (35.0)
Moderate	10 (66.7)	31 (43.7)	9 (60.0)	32 (53.3)
High	3 (20.0)	6 (8.5)	0 (0.0)	7 (11.7)
**Status, n (%)**				
Alive	12 (80.0)	52 (73.2)	9 (60.0)	45 (75.0)
Dead	3 (20.0)	19 (26.8)	6 (30.0)	15 (25.0)
**Right heart catheterization**				
mPAP, mmHg	51.8 ± 11.0	50.4 ± 14.9^c^	48.4 ± 16.1	44.6 ± 13.5
PAWP, mmHg	6.7 ± 3.1	7.3 ± 3.5	7.3 ± 3.3	7.0 ± 2.9
CO, L/min	4.0 ± 1.2^a^, ^b^	4.8 ± 1.3	5.1 ± 1.4	4.7 ± 1.2
CI, L/min/m^2^	2.5 ± 0.7^a^	3.0 ± 0.8	3.0 ± 0.6	2.8 ± 0.6
mRAP, mm Hg	7.4 ± 3.54	7.2 ± 3.0	6.5 ± 1.77	7.5 ± 3.0
PVR, mm Hg	13.0 ± 5.1^a,^ ^b^	10.1 ± 5.0	7.7 ± 3.4	8.6 ± 4.4
SvO_2_, %	62.9 ± 7.4^a^	67.7 ± 8.3^c^	62.1 ± 9.9	62.3 ± 7.4
**Cardiopulmonary exercise testing**				
Peak VO_2_/kg, mL/min/kg	13.5 ± 3.3	14.3 ± 4.1	13.0 ± 4.0	14.1 ± 4.5
Peak P_ET_O_2_, mmHg	128.9 ± 6.8^a^	124.9 ± 7.0^c^	129.9 ± 5.3	127.4 ± 6.3
Peak P_ET_CO_2_, mmHg	23.5 ± 7.0	26.6 ± 6.6^c^	22.2 ± 7.1	22.1 ± 8.4
Peak VO_2_/HR, L/min	5.0 ± 1.6^a^	5.9 ± 1.7	5.8 ± 1.4	6.0 ± 1.6
RER	1.1 ± 0.2	1.1 ± 0.5	1.1 ± 0.1	1.1 ± 0.6
VO_2_/VE, ml/L	15.9 ± 5.3^a^	20.8 ± 5.3^c^	17.6 ± 3.6	18.5 ± 4.8
VE/VCO_2_ slope, %pred	65.8 ± 29.1^a^	49.3 ± 15.1^c^	55.0 ± 12.6	57.1 ± 19.8
OUEP, ml/L	25.2 ± 5.4	27.4 ± 5.1^c^	23.5 ± 3.3	23.3 ± 4.7
Lowest VE/VCO_2_, %pred	49.0 ± 13.8	43.5 ± 10.1^c^	47.6 ± 10.1	51.6 ± 13.5
**Echocardiography**				
TAPSE, cm	1.7 ± 1.0	1.7 ± 0.3	1.8 ± 0.3	1.8 ± 0.3
RATD, cm	4.7 ± 1.2	4.4 ± 0.9	4.6 ± 1.0	4.7 ± 0.9
RALD, cm	5.3 ± 1.3	5.0 ± 0.8	5.2 ± 1.1	5.3 ± 0.9
RVEDTD, cm	4.6 ± 0.8^a,^ ^b^	4.0 ± 0.8	3.9 ± 0.5	4.2 ± 0.8
RVESTD, cm	6.3 ± 0.8	6.4 ± 0.9	6.7 ± 0.7	6.4 ± 0.7
ENDSEI	1.5 ± 0.2^b^	1.4 ± 0.3^c^	1.2 ± 0.2	1.2 ± 0.3
PASP, mmHg	80.5 ± 23.7	73.9 ± 27.0	67 ± 20.3	74.9 ± 25.3
**Targeted-PAH treatment**				
Ambrisentan + Sildenafil	6 (40.0)	20 (28.2)	7 (46.7)	27 (45.0)
Ambrisentan + Bosentan + Tadalafil	4 (26.7)	28 (39.4)	3 (20.0)	25 (41.7)
Macitentan + Riociguat + Tadalafil	5 (33.3)	23 (32.4)	5 (33.3)	8 (13.3)

Values are expressed as the mean ± SD, n (%), or median (interquartile range) and percentage of measured to predicted values (%pred). ^a^*P* < 0.05, IPAH with EIS vs. IPAH without EIS. ^b^*P* < 0.05, IPAH with EIS vs. CTEPH with EIS. ^c^*P* < 0.05, IPAH without EIS vs. CTEPH without EIS. Abbreviations as in Table 1.

### Comparisons of idiopathic pulmonary arterial hypertension and chronic thromboembolic pulmonary hypertension stratified by exercise-induced venous-to-systemic shunt

**FIGURE 2:**
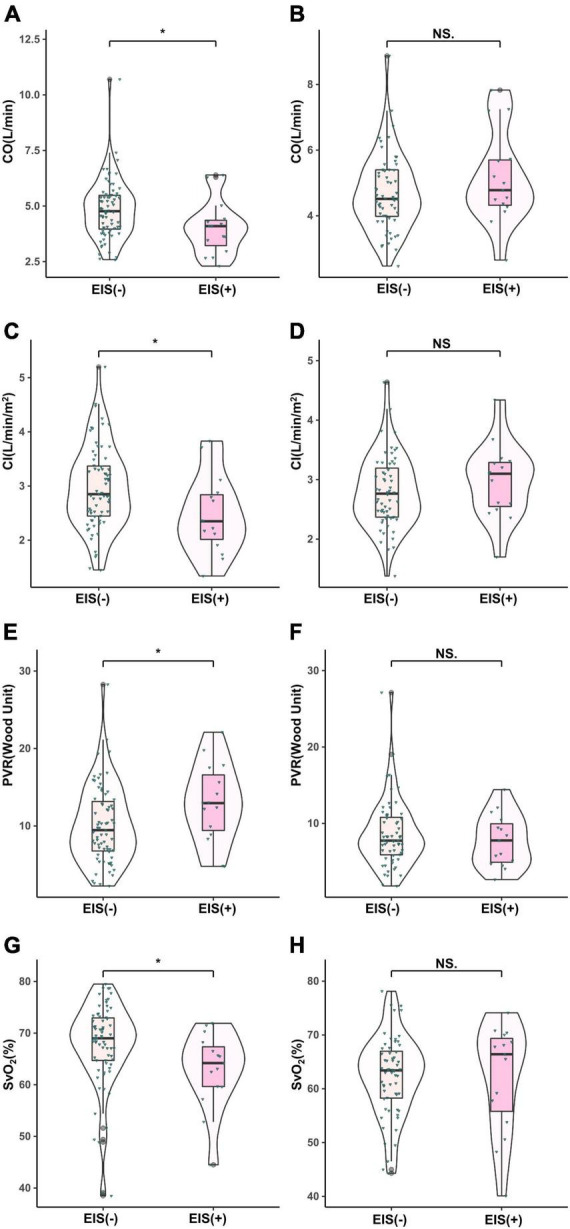
Comparison of hemodynamic parameters in IPAH and CTEPH patients stratified by EIS (+). *P < 0.05. IPAH, idiopathic pulmonary arterial hypertension; CTEPH, chronic thromboembolic pulmonary hypertension; EIS, exercise-induced right-to-left shunt; CO, cardiac output; CI, cardiac index; PVR, pulmonary vessel resistance; SvO_2_, mixed venous oxygen saturation. **(A,C,E,F)**, IPAH; **(B,D,G,H)**, CTEPH.

**FIGURE 3:**
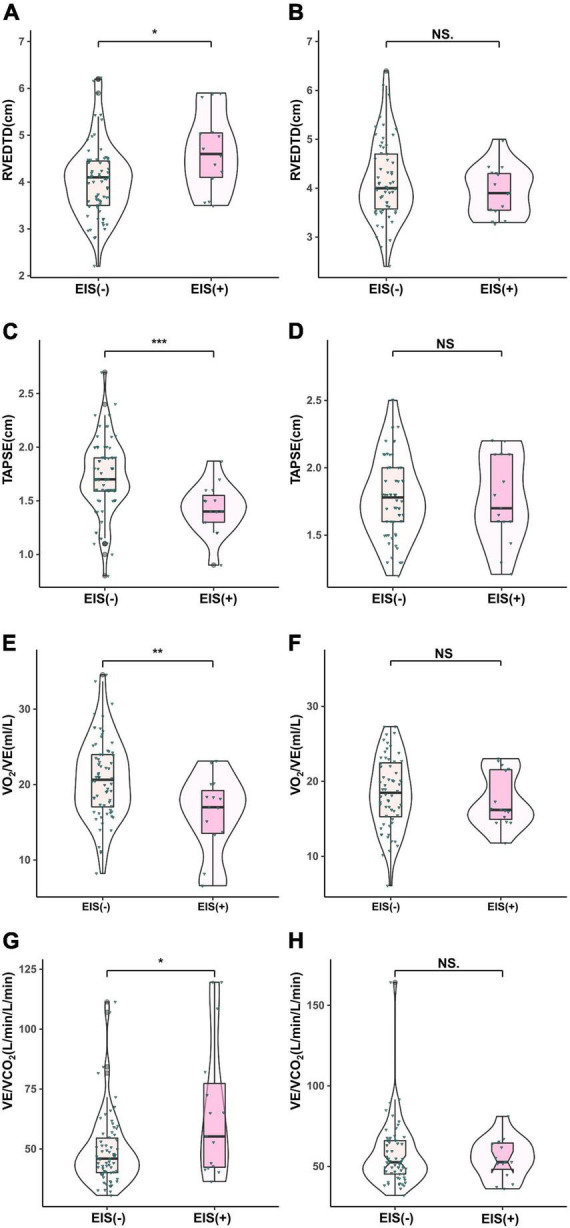
Comparison of echocardiographic parameters in IPAH **(A,C,E,F)** and CTEPH **(B,D,G,H)** patients stratified by EIS (+). **P < 0.01, ***P < 0.001. IPAH, idiopathic pulmonary arterial hypertension; CTEPH, chronic thromboembolic pulmonary hypertension; EIS, exercise-induced right-to-left shunt; RVEDTD, right ventricular end-diastolic transverse diameter; TAPSE, tricuspid annular plane systolic excursion; VCO_2_, carbon dioxide output; VE, minute ventilation; VO_2_, oxygen uptake.

With regard to hemodynamics, CO, CI, and SvO_2_ decreased among EIS (+) IPAH patients, while PVR increased significantly. No differences were found in CO, CI, SvO_2_, or PVR between the CTEPH patients, irrespective of the presence of EIS (Table 2 and Figure 2). Table 2 and Figure 3 depict the differences in exercise tolerance, ventilation efficiency, right heart function and submaximal exercise tolerance among EIS (+) or EIS (−) IPAH and CTPEH patients. Among IPAH patients, neither peak VO_2_ nor peak VO_2_/HR, which represented exercise tolerance and heart function, respectively, distinguished EIS (+) from EIS (−) patients. In terms of ventilator efficiency, VE/VCO_2_ was higher in EIS (+) IPAH patients than in EIS (−) IPAH patients, suggesting more impaired ventilation efficiency (65.8 ± 29.1 L/min/L/min vs. 49.3 ± 15.1 L/min/L/min, *P* < 0.05). However, other parameters reflecting ventilation efficiency, such as peak P_ET_O_2_, peak P_ET_CO_2_, and lowest VE/VCO_2_, did not exhibit differences. With regard to submaximal exercise tolerance, VO_2_/VE was lower in EIS (+) IPAH patients than in EIS (−) IPAH patients (15.9 ± 5.3 ml/L vs. 20.8 ± 5.3 ml/L, *P* < 0.05), while OUEP was not different. Among the echocardiography parameters, TAPSE decreased, while RVEDTD increased significantly among both EIS (+) and EIS (−) IPAH patients (4.6 ± 0.8 cm vs. 4.0 ± 0.8 cm, *P* < 0.05). Neither echocardiography nor CPET overall mean parameters could distinguish CTEPH patients with and without EIS.

### Influencing factors associated with exercise-induced venous-to-systemic shunt in idiopathic pulmonary arterial hypertension and chronic thromboembolic pulmonary hypertension patients

First, stratified by PH category, the risk, CI, CO, peak P_ET_O_2_, peak VO_2_/HR, VO_2_/VE, VE/VCO_2_, and RVEDTD achieved statistical significance in binary univariate logistical analysis. Then, excluding the influence of collinearity between variables, binary multivariate logistical analyses with the stepwise method were performed, which revealed that VO_2_/VE was significantly associated with EIS (+) in IPAH patients (odds ratio: 0.833, 95% CI: 0.736–0.943, *P* < 0.004) (Table 3). No significant variable was found to be associated with EIS (+) CTEPH patients.

**TABLE 3:** Binary logistic regression analysis of factors correlated with EIS in IPAH patients.

Variables	Univariate			Multivariate		
	β	OR (95% CI)	*P*-value	β	OR (95% CI)	*P*-value
Risk	−2.140	0.118 (0.016, 0.859)	0.035			
CI, L/min/m^2^	−1.011	0.364 (0.151, 0.943)	0.024			
CO, L/min	−0.606	0.545 (0.316, 2.102)	0.030			
Peak P_ET_O_2_, mmHg	0.087	1.090 (1.001, 1.188)	0.048			
Peak VO_2_/HR, ml/beat	−0.395	0.674 (0.455, 0.998)	0.049			
VO_2_/VE, ml/L	−0.183	0.833 (0.736, 0.943)	0.004	−0.183	0.833 (0.736, 0.943)	0.004
VE/VCO_2_, L/min/L/min	0.036	1.037 (1.010, 1.064)	0.007			
RVEDTD, cm	0.905	2.473 (1.221, 5.009)	0.012			

β, regression coefficient; SE (β), standard error of the regression coefficient; *OR*, odds ratio; 95% *CI*, 95% confidence interval. CI, cardiac index; CO, cardiac output; IPAH, idiopathic pulmonary arterial hypertension; Peak PETO_2_, peak end-tidal partial pressure of O_2_; Peak VO_2_/HR, peak oxygen pulse; RVEDTD, right ventricular end-diastolic transverse diameter; VCO_2_, carbon dioxide output; VE, minute ventilation; VO_2_, oxygen uptake.

### Impact of all-cause mortality stratified by exercise-induced venous-to-systemic shunt in idiopathic pulmonary arterial hypertension patients

**FIGURE 4:**
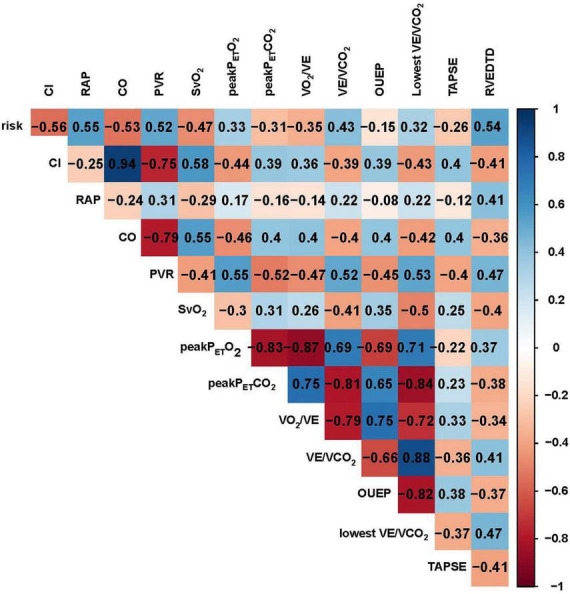
Correlation analysis of demographics, CPET, hemodynamics and echocardiography in patients with IPAH. CO, cardiac output; CI, cardiac index; PVR, pulmonary vessel resistance; SvO_2_, mixed venous oxygen saturation; RAP, right atrial pressure; Peak P_ET_CO_2_, peak end–tidal partial pressure of CO_2_, Peak P_ET_O_2_, peak end–tidal partial pressure of O_2_; VCO_2_, carbon dioxide output; VE, minute ventilation; VO_2_, oxygen uptake; RVEDTD, right ventricular end-diastolic transverse diameter; TAPSE, tricuspid annular plane systolic excursion; OUEP, oxygen uptake efficiency plateau.

**FIGURE 5:**
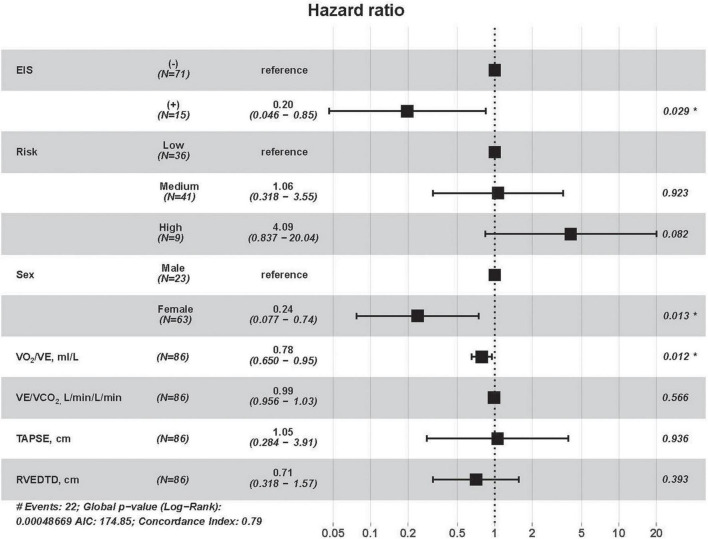
Cox univariate regression analyses for all-cause mortality among IPAH patients. RVEDTD, right ventricular end-diastolic transverse dimension; TAPSE, tricuspid annular plane systolic excursion; EIS, exercise-induced right-to-left shunt; VCO_2_, carbon dioxide output; VE, minute ventilation; VO_2_, oxygen uptake; IPAH, idiopathic pulmonary arterial hypertension.

All-cause mortality occurred in 43 (26.7%) patients during a median follow-up of 6.5 (4.7, 7.4) years. The follow-up rate was 100%. In 86 IPAH patients, all-cause mortality occurred in 22 patients. Based on high correlation coefficients (Figure 4), we excluded those parameters with strong collinearity. In addition, considering that the low, intermediate and high simplified PH risk stratifications were grouped by NT-proBNP, WHO FC, RAP, CI, SvO_2_, and 6MWD, we retained the simplified risk stratification and excluded the abovementioned dimensional parameters. Moreover, based on clinical significance and significant differences, EIS, risk, sex, VO_2_/VE, VE/VCO_2_, TAPSE, RVEDTD, and ENDSEI were selected for multivariate Cox regression (Figure 5). The predictors of all-cause mortality in the IPAH cohort were EIS, sex, VO_2_/VE, and RVEDTD with a cutoff of 20.99 ml/L. In CTEPH patients, none of the above parameters were independent influencing factors of EIS (Supplementary Figure 1).

**FIGURE 6:**
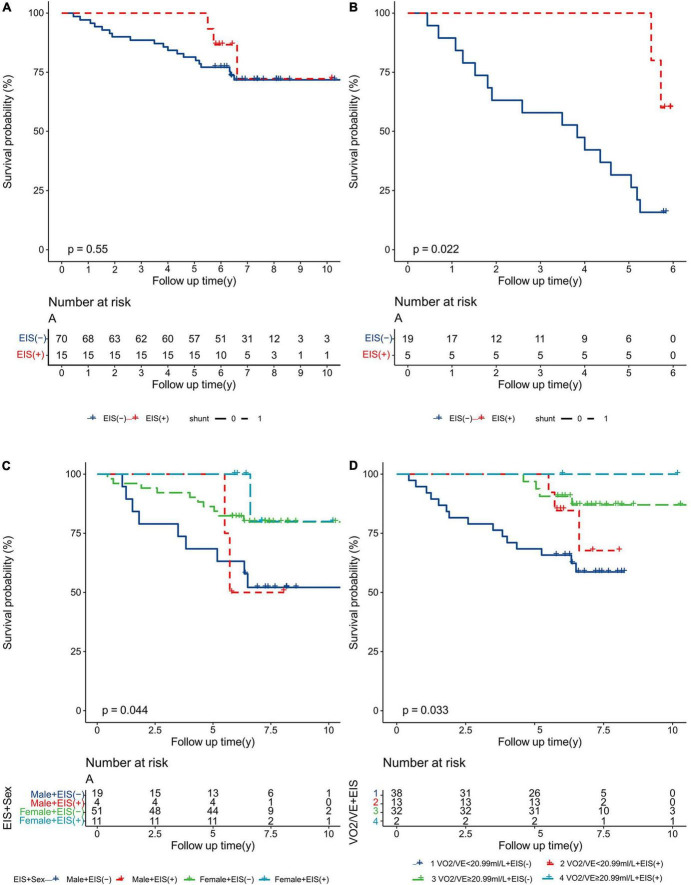
Kaplan–Meier analysis for all-cause mortality in IPAH patients, separated by **(A)** 10-year survival; **(B)** 6-year survival; **(C)** sex and EIS; and **(D)** VO_2_/VE and EIS. EIS, exercise-induced right-to-left shunt; VE, minute ventilation; VO_2_, oxygen uptake; IPAH, idiopathic pulmonary arterial hypertension.

Kaplan–Meier survival curves and log-rank tests were used to identify scores that would predict survival outcomes. Figure 6A shows no differences in 10-year survival between EIS (+) and EIS (−) IPAH patients; however, EIS (+) IPAH patients had better 6-year survival than EIS (−) patients (Figure 6B). Figures 6C,D show the Kaplan–Meier survival estimates for EIS with sex and VO_2_/VE, respectively. Female EIS (+) IPAH patients had a better prognosis than male EIS (−) IPAH patients; IPAH patients with VO_2_/VE ≥ 20.99 ml + EIS (+) had a better prognosis than those with VO_2_/VE < 20.99 ml and EIS (−).

**FIGURE 7:**
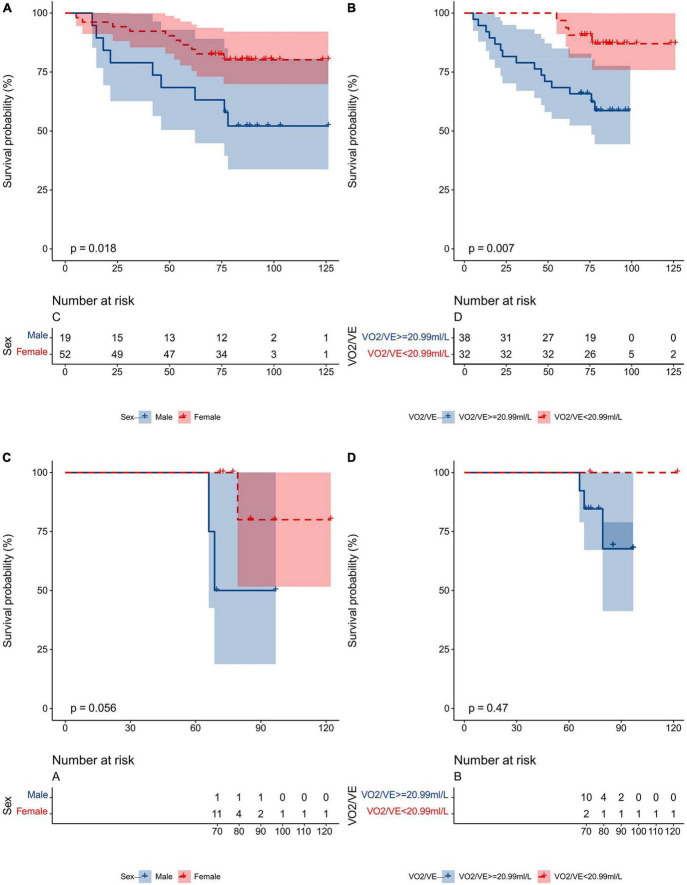
Kaplan–Meier analysis for all-cause mortality in IPAH patients, separated by **(A)** sex in the EIS (–) subgroup; **(B)** VO_2_/VE (<20.99 ml/L vs. ≥20.99 ml/L) in the EIS (–) subgroup; **(C)** sex in the EIS (+) subgroup; **(D)** VO_2_/VE (<20.99 ml/L vs. ≥20.99 ml/L) in the EIS (+) subgroup. EIS, exercise-induced right-to-left shunt; VE, minute ventilation; VO_2_, oxygen uptake; IPAH, idiopathic pulmonary arterial hypertension.

For EIS (−) IPAH patients, the prognosis of females was significantly better than that of males (Figure 7A); the prognosis of patients with VO_2_/VE ≥ 20.99 ml/L was much better than that of those with VO_2_/VE < 20.99 ml/L (Figure 7B). For EIS (+) IPAH patients, there were no significant differences regardless of sex or VO_2_/VE (Figures 7C,D).

However, for CTEPH patients, there were no significant prognoses regardless of 10-year survival, 1-year survival, 5-year survival, and 6-year survival (Supplementary Figures 2A–D, respectively).

## Discussion

Our study found that the relationship between EIS and disease severity or prognosis was significantly different in IPAH and CTEPH patients. EIS (+) IPAH patients had worse peak end-tidal O_2_, VO_2_/VE and TAPSE than EIS (−) IPAH patients. OUE, namely VO_2_/VE, was an independent factor of EIS among IPAH patients. IPAH patients with EIS, higher VO_2_/VE and female sex had better survival. Both EIS (+) and EIS (−) CTEPH patients had similar PAH severity and prognosis.

In our study, the incidence of EIS in patients with PH was 18.6%, while the incidence of EIS in the IPAH and CTEPH groups was 17.4 and 20.0%, respectively, which were lower than the rates previously reported (7, 9). There may be some reasons. We did not include discordant EIS patients in our study. In addition, in our study, some patients had undergone targeted-PAH treatment, which reduced their PVR compared with the baseline level, thus reducing the likelihood of EIS during CPET. The incidence of EIS in IPAH and CTEPH patients was similar, but the correlation with disease severity and prognosis varied. We infer that the different pathophysiological mechanisms of CTEPH and IPAH might be one of the causes. In addition to microarterial lesions similar to those in IPAH patients, CTEPH patients have thrombi in the main trunk, leaves, segments, subsegments and other parts.

In our study, peak P_ET_O_2_ significantly increased in patients with EIS (+) PH, while VO_2_/VE and TAPSE significantly decreased in EIS (+) PH patients. When EIS occurs, oxygen-deficient blood with high concentrations of CO_2_ and H^+^ is shunted into the systemic circulation, which stimulates chemoreceptors in the arteries to maintain the arterial homeostasis of H^+^ and PaCO_2_ (26). During this process, alveolar ventilation increases acutely, resulting in a rapid increase in PaO_2_ and a rapid decrease in PaCO_2_ in the alveoli, which is reflected in the increase in peak P_ET_O_2_ and the decrease in peak P_ET_CO_2_ (9). Unfortunately, we did not find a significant difference in peak P_ET_CO_2_ between EIS (+) and EIS (−) patients. In addition, VO_2_/VE represents the OUE of the pulmonary artery to a certain extent (27). Therefore, the decrease in VO_2_/VE in EIS patients indicates that such patients have lower OUE. OUE has been widely used to evaluate the severity and prognosis of cardiopulmonary diseases such as chronic heart failure, coronary heart disease and PH (28–30), and also be used to evaluate submaximal exercise endurance in patients with PH (31). EIS (+) patients have lower OUE, which we speculate may be explained as follows. First, patients with shunts have poorer hemodynamics, and experience anaerobic exercise earlier (7), which can lead to lactate buildup. The acid and hypoxia in the shunt blood can significantly stimulate ventilation to maintain homeostasis in the arteries, which can cause patients to experience unbearable dyspnea and to eventually be forced to stop exercising, resulting in a decrease in oxygen uptake. Second, a combination of acute alveolar hyperventilation and reduced right ventricular blood flow into the pulmonary vasculature in EIS (+) patients can lead to severe V/Q dysregulation that ultimately hinders the patient’s gas exchange. Furthermore, TAPSE reflects the longitudinal systolic function of the right ventricular myocardium (32). Thus, a lower TAPSE may predict reduced right ventricular function in EIS (+) patients.

We found the differences in clinical data between EIS (+) and EIS (−) IPAH and CTEPH patients and the influencing factors of EIS according to different PH classifications. We found that there was no significant difference in hemodynamics, CPET or echocardiography for the occurrence of EIS in CTEPH patients, but the situation was significantly different in IPAH patients. In terms of hemodynamics, EIS (+) IPAH patients had lower CO, CI, and SvO_2_ and higher PVR levels than EIS (−) IPAH patients, suggesting that EIS (+) patients may have more severe PH, which would be in line with the results of Guo et al. (7). In addition, EIS (+) IPAH patients had lower TAPSE and higher RVTDED than EIS (−) patients on echocardiography, which further indicated that such patients had worse right heart function. The higher VE/VCO_2_ indicated that the EIS (+) IPAH patients had a more impaired ventilation efficiency, with a sharp increase in lung ventilation during CPET. The VE/VCO_2_ ratio is the result of both the dead space to tidal volume ratio (VD/VT) and the PaCO_2_ set point. When EIS occurs, diverting deoxygenated, acidemic, and CO_2_-rich blood stimulate arterial chemoreceptors, causing an immediate increase in ventilation, as manifested by rapid increases in alveolar PaO_2_ and decreases in PaCO_2_.

The remodeling of the pulmonary vasculature in IPAH patients and the occlusion of small pulmonary vessels in CTEPH patients both lead to an increase in PVR. Moreover, EIS (+) patients have reduced blood flow from the right ventricle to the pulmonary vasculature, which further results in alveolar hypoperfusion and an increase in VE/VCO_2_ (28, 33). Notably, we found that VO_2_/VE in EIS (+) IPAH patients was significantly lower than that in EIS (−) IPAH patients, and VO_2_/VE was an independent factor influencing whether IPAH patients developed EIS. This suggests that OUE may indicate a more serious disease in IPAH patients and that paying attention to patients’ OUE may be helpful for the timely detection of such IPAH patients.

In the follow-up, all-cause mortality stratified by EIS was assessed in the IPAH group. We found that male sex and lower OUE predicted worse survival in IPAH patients, especially in those with EIS (+). In EIS (−) IPAH patients, although male sex and lower OUE represented a trend of lower survival, they were not statistically significant, which we speculate might be related to the sample size. Interestingly, despite more severe hemodynamic characteristics (lower CO, CI, and SvO_2_), patients with EIS and IPAH had better survival at 6 years. This might be because the major cause of death in patients with PAH and CTEPH was right heart failure, and the possibility of a right-to-left shunt allowed the unloading of the right side of the heart. Consistent with our study, Ronald et al. (10) noted that reduced aerobic capacity and ventilation efficiency were associated with increased mortality in PAH patients. In addition, Tang et al. (34) confirmed that OUE has good prognostic value in IPAH patients. The reasons may be as follows: on the one hand, hyperventilation during the occurrence of EIS causes OUE (namely, VO_2_/VE) to decline; on the other hand, OUE is related to the patient’s cardiac function. Poor cardiac function affects the pumping ability of the heart, namely, cardiac output, leading to a decrease in OUE (35). In addition, VO_2_ is dependent both on the cardiac output and the delivery of oxygen (QxCaO_2_) as well as oxygen extraction in the tissue (CaO_2_–CvO_2_), which is of importance since in EIS, there is oxygen desaturation and impaired oxygen delivery (QxCaO_2_). Therefore, OUE can better reflect the physiological changes in the disease and the severity of the harm caused by the disease. We also found that male EIS (+) IPAH patients had lower survival rates, which suggests that sex differences in changes in venous-to-systemic circulation shunts may contribute to further analysis of independent clinical outcomes.

Interestingly, by stratifying IPAH or CTEPH by EIS and comparing the relevant clinical data and outcomes, we found that EIS seems to have a greater impact on IPAH patients, which has not been previously studied. The reasons for this finding may be as follows: First, this may be related to the low incidence of EIS in this study, which resulted in some comparisons having clinically relevant trends but no significant differences. Second, the two classifications of disease are treated differently, and the clinical orientation of targeted drugs for IPAH and CTEPH may be inconsistent, which results in different pulmonary vascular responses to exercise-induced elevated PVR in the two PH classifications. Third, it could be associated with the pathophysiological manifestations of the two types of PH. The pathological changes in IPAH mainly involve the distal pulmonary arterioles, while CTEPH involves both the pulmonary arterioles and the larger pulmonary arteries, which may lead to the different reactions to EIS.

### Study limitations

There are several limitations to our study. First, it only included patients with IPAH and CTEPH, while ignoring other classifications of PH, which added some bias to the study. Future studies should include various types of PH. Second, we did not compare changes in CPET parameters from rest to the end of unloading, as previous studies have done, which may also be related to PH severity. Finally, this was a small, single-center, retrospective study. A large, multicenter, prospective study may be needed to better evaluate the condition and prognosis of PH through relevant indicators.

## Conclusion

EIS exhibits different profiles among IPAH and CTEPH patients. The association between EIS and disease severity or prognosis is quite different in IPAH and CTEPH patients, which may be related to the pathophysiological characteristics of the two diseases and requires special attention in clinical practice. IPAH patients with EIS have worse peak end-tidal O_2_, VO_2_/VE and TAPSE than those without EIS. VO_2_/VE is an independent factor of EIS among IPAH patients. IPAH patients with EIS, higher VO_2_/VE and female sex have better survival, which indicates that there is a greater association between EIS and OUE, but no such association is found in CTEPH patients. However, further large-sample randomized controlled trials are needed to confirm and explore the specific mechanisms involved. In addition, this study may provide new ideas for further research on the pathophysiological mechanisms of the two types of PH.

## Data availability statement

The raw data supporting the conclusions of this article will be made available by the authors, without undue reservation.

## Ethics statement

The studies involving human participants were reviewed and approved by the Institutional Ethics Committee of Shanghai Pulmonary Hospital Local Committees. The patients/participants provided their written informed consent to participate in this study.

## Author contributions

RJ, JG, and J-ML contributed to the conception and design of the study. PY, X-XS, and Z-YG organized the database. H-LQ, C-JL, and Q-HZ performed the statistical analysis. RJ and SW wrote the first draft of the manuscript. S-GG, LW, JH, RZ, H-TL, W-HW, and LW wrote sections of the manuscript. All authors contributed to manuscript revision, read, and approved the submitted version.

## Footnotes

1
http://www.r-project.org

## Supplementary material

Supplementary Figure 1 Cox univariate regression analyses for all-cause mortality among CTEPH patients. RVEDTD, right ventricular end-diastolic transverse dimension; TAPSE, tricuspid annular plane systolic excursion; EIS, exercise-induced right-to-left shunt; VCO_2_, carbon dioxide output; VE, minute ventilation; VO_2_, oxygen uptake.

Supplementary Figure 2 Kaplan–Meier analysis for all-cause mortality in CTEPH patients, separated by **(A)** 10-year survival, **(B)** 1-year survival, **(C)** 5-year survival, and **(D)** 6-year survival. CTEPH, chronic thromboembolic pulmonary hypertension.
