# Supplementary figures and images for: Differences in disease severity and prognosis of exercise-induced right-to-left shunt between idiopathic pulmonary arterial hypertension and chronic thromboembolic pulmonary hypertension patients
Source: Front Cardiovasc Med. 2022 Dec 12;9:976730. doi: 10.3389/fcvm.2022.976730 (PMC9791184; doi:10.3389/fcvm.2022.976730)

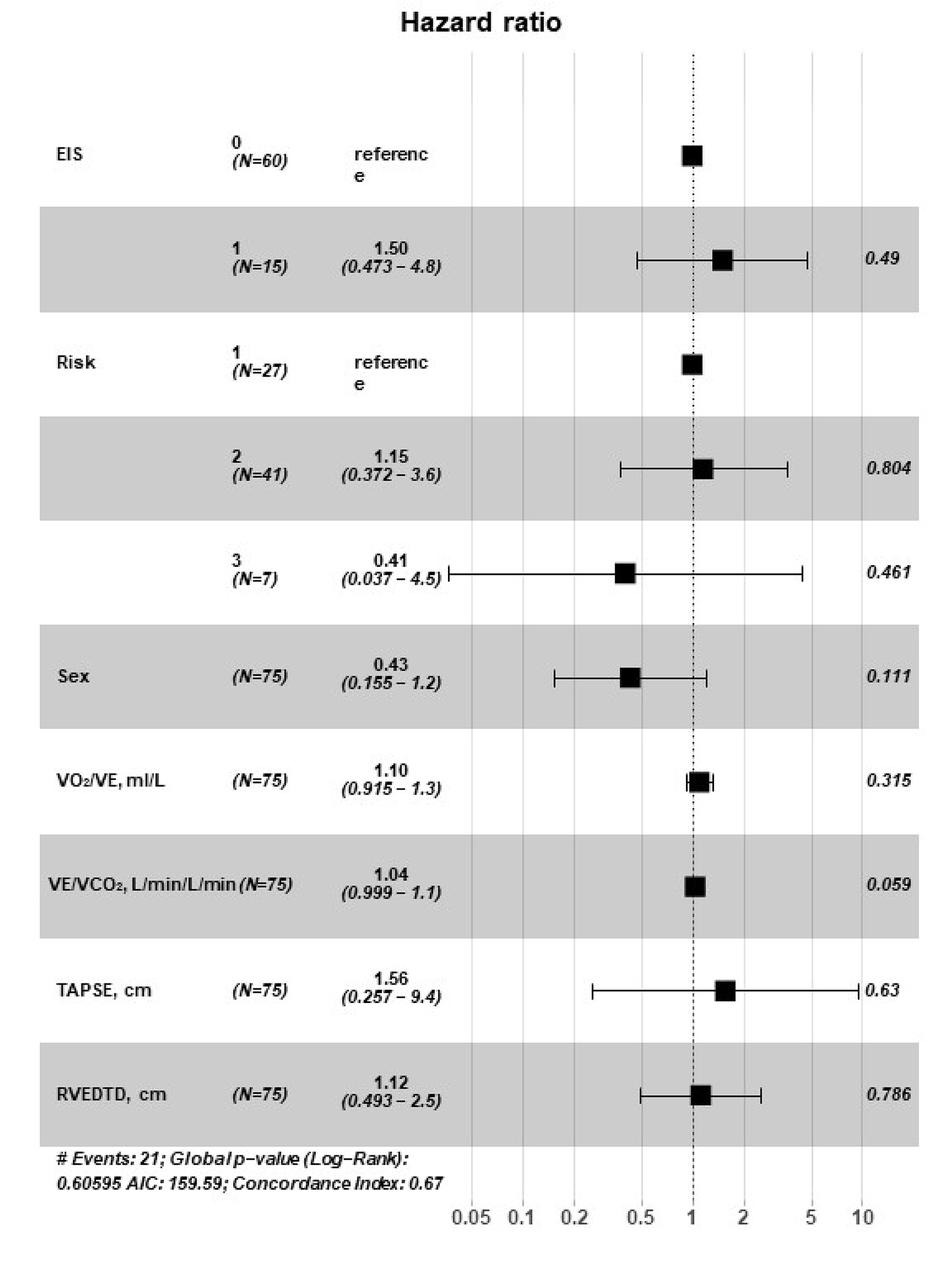

Supplement: Supplementary Figure 1 — Cox univariate regression analyses for all-cause mortality among CTEPH patients. RVEDTD, right ventricular end-diastolic transverse dimension; TAPSE, tricuspid annular plane systolic excursion; EIS, exercise-induced right-to-left shunt; VCO2, carbon dioxide output; VE, minute ventilation; VO2, oxygen uptake. [file Image_1.TIF]

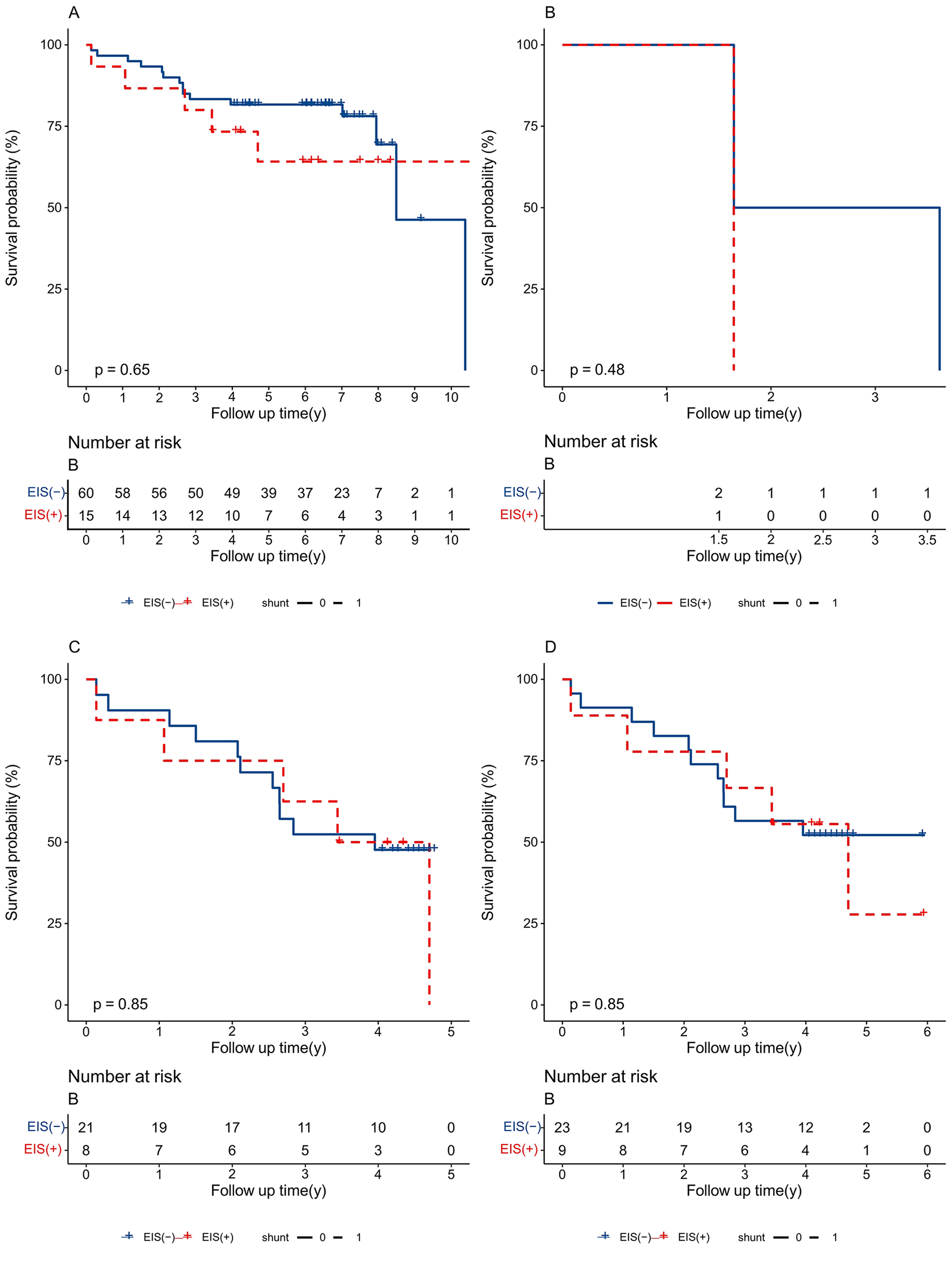

Supplement: Supplementary Figure 2 — Kaplan–Meier analysis for all-cause mortality in CTEPH patients, separated by (A) 10-year survival, (B) 1-year survival, (C) 5-year survival, and (D) 6-year survival. CTEPH, chronic thromboembolic pulmonary hypertension. [file Image_2.TIF]
